# Association of cardiometabolic multimorbidity and adherence to a healthy lifestyle with incident dementia: a large prospective cohort study

**DOI:** 10.1186/s13098-023-01186-8

**Published:** 2023-10-24

**Authors:** Sizheng Xiong, Ningxin Hou, Feifei Tang, Jun Li, Hongping Deng

**Affiliations:** 1 Department of Vascular Surgery, Renmin Hospital of Wuhan University, Wuhan, 430060 China; 2 Division of Cardiovascular Surgery, Tongji Hospital, Tongji Medical College, Huazhong University of Science and Technology, Wuhan, China; 3 Department of Cardiovascular Surgery, Central Hospital of Wuhan, Huazhong University of Science and Technology, Wuhan, China

**Keywords:** Cardiometabolic Disease, Multimorbidity, Lifestyle, Dementia, Brain volume

## Abstract

**Background:**

The co-occurrence of cardiometabolic diseases (CMDs) is increasingly prevalent and has been associated with an additive risk of dementia in older adults, but the extent to which this risk can be offset by a healthy lifestyle is unknown. We aimed to examine the associations of cardiometabolic multimorbidity and lifestyle with incident dementia and related brain structural changes.

**Methods:**

This prospective study extracted health and lifestyle data from 171 538 UK Biobank participants aged 60 years or older without dementia at baseline between 2006 and 2010 and followed up until July 2021, as well as brain structural data in a nested imaging subsample of 11 972 participants. Cardiometabolic multimorbidity was defined as the presence of two or more CMDs among type 2 diabetes, coronary heart disease, stroke, and hypertension. Lifestyle patterns were determined based on 7 modifiable lifestyle factors including smoking, alcohol consumption, physical activity, diet, sleep duration, sedentary behavior, and social contact.

**Results:**

Over a median follow-up of 12.3 years, 4479 (2.6%) participants developed dementia. The presence of CMDs was dose-dependently associated with an increased risk of dementia. Compared with participants with no CMDs and a favourable lifestyle, those with ≥ 3 CMDs and an unfavourable lifestyle had a five times greater risk of developing dementia (HR 5.33, 95% CI 4.26–6.66). A significant interaction was found between CMD status and lifestyle (*P*_interaction_=0.001). The absolute difference in incidence rates of dementia per 1000 person years comparing favourable versus unfavourable lifestyle was − 0.65 (95% CI − 1.02 to − 0.27) among participants with no CMDs and − 5.64 (− 8.11 to − 3.17) among participants with ≥ 3 CMDs, corresponding to a HR of 0.71 (0.58–0.88) and 0.42 (0.28–0.63), respectively. In the imaging subsample, a favourable lifestyle was associated with larger total brain, grey matter, and hippocampus volumes across CMD status.

**Conclusion:**

Our findings suggest that adherence to a healthy lifestyle might substantially attenuate dementia risk and adverse brain structural changes associated with cardiometabolic multimorbidity.

**Supplementary Information:**

The online version contains supplementary material available at 10.1186/s13098-023-01186-8.

## Background

Type 2 diabetes, coronary heart disease (CHD), and stroke are well-established cardiometabolic risk factors for dementia [1–4]. With the ageing of the population, the prevalence of cardiometabolic multimorbidity, defined as two or more cardiometabolic diseases (CMDs), is rising rapidly in older adults [5]. Concurrent CMDs have been shown to interact with each other to increase the risk of negative outcomes [6]. Although relevant evidence about dementia is overall sparse, two recent studies have revealed dose-dependent associations between CMDs and dementia incidence or progression [7, 8], indicating that people with cardiometabolic multimorbidity are at high risk of developing dementia and concerted efforts to reduce the risk are imperative.

Because of limited value of pharmacological therapies currently used for treating dementia, directing the focus toward primary prevention strategies has become a high priority. Healthy lifestyle factors, including nonsmoking, moderate alcohol consumption, physical activity, and healthy diet, have been individually associated with a lower risk of dementia [9]. These lifestyle behaviours often cluster in people and may have synergistic effects on dementia risk [10, 11]. Additionally, mounting evidence has emphasised a role for emerging lifestyle factors, such as sleep duration, television viewing time, and social contact [12–14]. Since knowledge about lifestyle-related risk factors of dementia is improving, incorporating the emerging factors in overall lifestyle scores will provide more useful information for public health intervention.

Lifestyle modification is a fundamental component for management of CMDs. A recent study reported that engagement in leisure activities and a rich social network conferred a reduced risk of dementia among older adults with CMDs [15]. However, no previous studies have identified whether and the extent to which the increased dementia risk associated with cardiometabolic multimorbidity can be offset by a broad combination of healthy lifestyle factors. Meanwhile, CMDs have been linked to brain damage before the clinical symptoms of cognitive impairment and dementia [16, 17], but the impact of a healthy lifestyle on brain structural changes in patients with CMDs, particularly those with cardiometabolic multimorbidity, remains unclear. Understanding such features could help tailor future interventions for dementia prevention among the high-risk individuals. In this study, we used data from the UK Biobank cohort to examine how a healthy lifestyle, which incorporates conventional and emerging lifestyle factors, affects the associations of cardiometabolic multimorbidity with incident dementia and brain structural measures. We hypothesised that adherence to a healthy lifestyle may counteract the risk of dementia among people with cardiometabolic multimorbidity.

## Methods

### Study design and population

The UK Biobank is a population-based cohort that recruited more than 500 000 participants aged 40–70 years who attended one of 22 assessment centres across the UK between 2006 and 2010 [18]. Participants completed touch-screen and nurse-led questionnaires, underwent physical measurements, and provided biological samples. A subgroup of participants received brain imaging tests between 2014 and 2020. In this study, analyses were restricted to individuals aged at least 60 years at baseline who had lifestyle information available, because most sporadic dementia occurs in older adults. Participants with prevalent cognitive impairment or dementia at baseline or a history of encephalitis, meningitis, amyotrophic lateral sclerosis, multiple sclerosis, head injury, or central nervous system infection were excluded from analyses. Those with type 1 diabetes or missing data on glycated hemoglobin (HbA_1c_) were also excluded. Disease diagnoses were based on a combination of self-report and hospital inpatient records.

The UK Biobank study has ethical approval from the North West Multi-Centre Research Ethics Committee (reference 11/NW/0382). All participants provided written informed consent for the study.

### Ascertainment of cardiometabolic diseases and multimorbidity

CMDs that included type 2 diabetes, CHD, stroke, and hypertension were ascertained at baseline using self-reported diagnoses and hospital inpatient records (UK Biobank codes are listed in Supplementary Table 1). The algorithms for identification of prevalent diabetes based on self-reported medical history and medications have been previously described [19]. Undiagnosed diabetes was defined as HbA_1c_ ≥ 48.0 mmol/mol (6.5%), in accordance with the American Diabetes Association criteria [20]. Hypertension was defined as previous professional diagnosis, taking blood pressure-lowering medications, systolic blood pressure ≥ 140 mm Hg, or diastolic blood pressure ≥ 90 mm Hg. CMD status was identified for each participant by summing the number of CMDs (diabetes, CHD, stroke, and hypertension) and categorised as no CMDs, 1 CMD, 2 CMDs, or ≥ 3 CMDs.

### Healthy lifestyle categories

We considered seven modifiable lifestyle behaviours known to be associated with dementia risk [9], including smoking, physical activity, alcohol consumption, diet, sleep duration, sedentary behaviour, and social contact, to generate a lifestyle score. All lifestyle data were derived from questionnaire responses at baseline. Details of the assessments of individual lifestyle factors can be found in Supplementary Table 2. Given that weight change is a common phenotype during CMD progression and is affected by medical treatment [21], body mass index (BMI) may not serve as a valid measure for weight management to be included in the lifestyle score.

We determined healthy and unhealthy categories for each lifestyle factor using national guidelines if available. Participants scored 1 point for each of healthy lifestyle factors: no current smoking, moderate alcohol consumption (up to 1 drink [14 g] per day for women and up to 2 drinks [28 g] per day for men) [22], regular physical activity (≥ 150 min per week of moderate activity or ≥ 75 min per week of vigorous activity) [23], a healthy diet pattern (adherence to at least 4 of the 7 endorsed dietary habits—consumption of an increased amount of fruits, vegetables, whole grains, fish, and a reduced amount of refined grains, processed and unprocessed meats) [24], adequate sleep duration (7–9 h/day), less sedentary behaviour (< 4 h per day of television viewing time) [25], frequent social contact (at least 2 of the 3 predefined characteristics—living with other people, regular friends or family visits, and engagement in social activities) [26]. The sum of these factors constituted a final lifestyle score ranging from 0 to 7, with higher scores indicating a healthier lifestyle. This lifestyle score has been validated elsewhere [27]. We then categorised the lifestyle patterns as favourable (score 6–7), intermediate (score 4–5), or unfavourable (score 0–3) based on distribution of the lifestyle score. In sensitivity analysis, we constructed a weighted lifestyle score considering the relative risk for each lifestyle factor estimated from the full adjusted model that included all seven lifestyle factors: weighted lifestyle score = (β1× factor1 + β2 × factor2 +…+ β7 × factor7) × (7/sum of the β coefficients).

### Dementia diagnosis

Incident dementia was ascertained through linkage to hospital admission records and death registry data. Date and cause of hospital admissions were obtained from the Health Episode Statistics for England and Wales and the Scottish Morbidity Records for Scotland. Information on death was provided by the National Health Service Digital for England and Wales and the National Health Service Central Register for Scotland. Additional cases were retrieved from primary care records. Dementia diagnoses were documented using the International Classification of Diseases (ICD-9 and ICD-10) codes for Alzheimer’s disease, vascular dementia, and other dementia classifications (Supplementary Table 1).

### Brain imaging

Brain MRI data including high-resolution, T1-weighted, three-dimensional MPRAGE and T2-weighted FLAIR images were acquired on a standard Siemens Skyra 3T scanner with a 32-channel receive head coil. The imaging data were processed and released as imaging-derived phenotypes. Full details of the imaging protocol and processing pipeline have been previously described [28]. We used imaging summary statistics of volumes of total brain, grey matter, white matter hyperintensity (WMH), and hippocampus. Brain tissue volumes were normalized for head size based on the external surface of the skull [28]. Median absolute deviation was used to exclude extreme outliers [29].

### Covariates

Socioeconomic status was derived from the Townsend deprivation index (in quintiles), a composite measure combining information on car ownership, housing, owner occupation, and employment [30]. Ethnicity was self-reported and categorised into white, Asian, black, or other ethnic group. Educational attainment was self-reported and coded as an ordinal variable (college or university degree, A levels/AS levels, O levels/GCSEs, CSEs or equivalent, NVQ/HND/HNC or equivalent, or none of these). Depression was ascertained during a nurse-led interview at baseline. Apolipoprotein E (*APOE*) haplotypes were extracted from the genetic data and categorised as *APOE* ε4 carriers (any ε4 allele) and noncarriers. Cognitive function tests (e.g. reaction time test of symbol matching and visual pairs memory test) were completed through a touchscreen tool.

### Statistical analysis

Descriptive characteristics of the study population by CMD status were presented as percentage for categorical variables and means with SDs for continuous variables. Follow-up time was calculated from baseline to the date of dementia diagnosis, death, or the censoring date (30 July 2021), whichever occurred first. Cox proportional hazards models with age the time scale were used to estimate hazard ratios (HRs) of incident dementia associated with CMD status (no CMDs, 1 CMD, 2 CMDs, or ≥ 3 CMDs) and lifestyle category (favourable, intermediate, or unfavourable). Apart from overall CMD status, we calculated HRs for mutually exclusive groups according to specific CMDs, compared with the no CMDs group. To test our main hypothesis, we examined the association of the combination of CMD status and lifestyle category (12 categories with no CMDs and favourable lifestyle as reference) with incident dementia. Statistical interaction between CMD status and lifestyle category was tested by fitting an interaction term in the models. All analyses were adjusted for age, sex, ethnicity, education, socioeconomic deprivation, depression, *APOE* ε4 carrier status, and cognitive performance at baseline. When investigating the individual association with CMD status, models were additionally adjusted for lifestyle category, and vice versa. Missing covariate data were included as a separate category for categorical variables (all missing covariates < 1%). The proportional hazards assumption was checked using Schoenfeld residuals and satisfied. We also examined the association between lifestyle category and incident dementia within each CMD status. Absolute rate differences in dementia incidence across lifestyle categories were estimated, with participants in the unfavourable lifestyle category as the reference group.

Analyses of CMD status and lifestyle category alone or in combination and volumes of total brain, grey matter, WMH, and hippocampus were conducted using linear regression models with each brain measure as the outcome. Due to the positively skewed distribution, WMH volume was log-transformed before modeling. For each brain measure, interaction between CMD status and lifestyle category was tested using Bonferroni correction for multiple testing.

In supplementary analyses, we included additional covariates of total cholesterol, BMI, and HbA_1c_ to examine whether these upstream cardiometabolic risk markers could partly explain the associations between cardiometabolic multimorbidity, lifestyle, and dementia risk. We also evaluated the possible influence of disease treatments by further adjusting for medications for high blood pressure, high cholesterol, and diabetes. Moreover, we used proportional subdistribution hazards regression models to account for the competing risk of death. Further sensitivity analyses were conducted by using imputed data for missing values of lifestyle factors and covariates, redefining low-risk alcohol consumption as no heavy drinking, and excluding dementia cases within the first 3 or 5 years of follow-up. Given the potential differences between participants with and without MRI data, analyses of associations with brain volumes were weighted by the inverse of their probability of being a complete data case. All statistical analyses were performed with SAS, version 9.4 (SAS Institute Inc). A two-sided *P* value < 0.05 was considered statistically significant.

## Results

### Population characteristics

Of the 502 505 participants recruited to UK Biobank, 217 489 (43.2%) were aged 60 years or older. After excluding participants with prevalent dementia (n = 236) or type 1 diabetes (n = 1044) at baseline, those with missing data on lifestyle metrics (n = 32 320) or HbA_1c_ (n = 10 470), or those fulfilled any other exclusion criteria (n = 1881), 171 538 participants were included in the analytic sample (Supplementary Fig. 1). The mean age of participants was 64.1 (SD 2.8) years and 88 389 (51.5%) were women. At baseline assessment, 46 368 (27.0%) participants were free of CMDs, and 100 640 (58.7%), 20 918 (12.2%), 3612 (2.1%) had 1, 2, and ≥ 3 CMDs, respectively. Characteristics of the participants by number of CMDs are presented in Table 1. On the basis of lifestyle score comprising seven behaviours, 25 472 (14.9%) participants were classified into the unfavourable lifestyle category, 90 102 (52.5%) into the intermediate lifestyle category, and 55 964 (32.6%) into the favourable lifestyle category. Participants with one or more CMDs had a lower proportion of favourable lifestyle than those with no CMDs.

**Table 1:** Baseline characteristics of participants by cardiometabolic disease status

	No CMDs	1 CMD	2 CMDs	≥ 3 CMDs
Participants	46 368	100 640	20 918	3612
Age, years	63.5 (2.8)	64.2 (2.8)	64.7 (2.9)	65.0 (2.9)
Women	28 390 (61.2)	51 792 (51.5)	7304 (34.9)	903 (25.0)
Body mass index, kg/m^2^	25.9 (3.8)	27.6 (4.3)	29.7 (5.0)	31.2 (5.2)
Ethnicity				
White	45 519 (98.5)	98 414 (98.1)	19 952 (95.6)	3368 (93.6)
Asian or Asian British	288 (0.6)	772 (0.8)	450 (2.2)	123 (3.4)
Black or Black British	104 (0.2)	459 (0.5)	231 (1.1)	49 (1.4)
Other ethnic group	320 (0.7)	701 (0.7)	230 (1.1)	58 (1.6)
Socioeconomic deprivation quintile				
1 (least deprived)	9783 (21.1)	20 628 (20.5)	3449 (16.5)	464 (12.8)
2	9284 (20.0)	20 666 (20.5)	3808 (18.2)	551 (15.2)
3	9329 (20.1)	20 378 (20.3)	3979 (19.0)	609 (16.9)
4	9239 (19.9)	20 078 (19.9)	4244 (20.3)	743 (20.6)
5 (most deprived)	8733 (18.8)	18 890 (18.8)	5438 (26.0)	1245 (34.5)
Education				
College or university degree	15 218 (33.1)	26 963 (27.0)	4537 (21.9)	605 (17.0)
A levels/AS levels	4664 (10.1)	9353 (9.4)	1710 (8.3)	294 (8.3)
O levels/GCSEs	9873 (21.5)	22 096 (22.1)	4104 (19.8)	651 (18.3
CSEs or equivalent	986 (2.1)	2207 (2.2)	490 (2.4)	102 (2.9)
NVQ/HND/HNC or equivalent	2851 (6.2)	7510 (7.5)	1881 (9.1)	359 (10.1)
None of the above	12 424 (27.0)	31 717 (31.8)	7984 (38.6)	1549 (43.5)
*APOE* ε4 carrier	12 702 (27.7)	27 754 (27.8)	5929 (28.5)	1009 (28.2)
Depression	2278 (4.9)	4125 (4.1)	1069 (5.1)	209 (5.8)
Healthy lifestyle factors				
No current smoking	42 314 (91.3)	93 544 (92.9)	19 066 (91.1)	3236 (89.6)
Moderate alcohol consumption	26 545 (57.2)	53 809 (53.5)	10 559 (50.5)	1686 (46.7)
Regular physical activity	26 803 (57.8)	57 155 (56.8)	10 656 (50.9)	1609 (44.5)
Healthy diet	25 648 (55.3)	51 612 (51.3)	9862 (47.2)	1541 (42.7)
Adequate sleep duration	35 395 (76.3)	76 268 (75.8)	15 173 (72.5)	2387 (66.1)
Less sedentary behavior	32 218 (69.5)	63 827 (63.4)	11 310 (54.1)	1659 (45.9)
Frequent social contact	42 733 (92.2)	92 649 (92.1)	18 787 (89.8)	3119 (86.3)
Lifestyle category				
Unfavourable	5654 (12.2)	14 421 (14.3)	4336 (20.7)	1061 (29.4)
Intermediate	23 329 (50.3)	53 489 (53.2)	11 387 (54.4)	1897 (52.5)
Favourable	17,385 (37.5)	32 730 (32.5)	5195 (24.8)	654 (18.1)

Data are n (%) or mean (SD). Percentages might not add to 100% due to rounding. A, Advanced; *APOE* ε4, apolipoprotein E4 allele; AS, Advanced Subsidiary; CMD, cardiometabolic disease; CSE, Certificate of Secondary Education; GCSE, General Certificate of Secondary Education; HNC, Higher National Certificate; HND, Higher National Diploma; NVQ, National Vocational Qualification; O, Ordinary

### Independent association of CMDs and lifestyle with dementia risk

Over a median follow-up of 12.3 years (IQR 11.5–13.0 years), 4479 (2.6%) incident cases of all-cause dementia were documented. Type 2 diabetes, CHD, and stroke were individually associated with dementia risk. The presence of concurrent CMDs was dose-dependently associated with an increased risk of dementia, and this association did not change after adjusting for lifestyle factors. Compared with the reference group with no CMDs, the HRs for dementia were 1.07 (95% CI 0.99–1.15), 1.74 (1.58–1.91), and 2.83 (2.46–3.25) for participants with 1, 2, and ≥ 3 CMDs, respectively (Table 2). In terms of lifestyle factors, each one was related to dementia risk with the exception of diet (Supplementary Table 3). There was a monotonic association between the number of healthy lifestyle factors and lower dementia risk (HR 0.89, 95% CI 0.87–0.91 per additional factor) (Supplementary Table 4). In multivariable models that included CMD status, participants with a favourable lifestyle had a HR for dementia of 0.67 (95% CI 0.61–0.73) compared with those who had an unfavourable lifestyle (Table 2).

**Table 2:** Risk of incident dementia in relation to cardiometabolic disease status and lifestyle category at baseline

	Total number of participants	Number of dementia cases	Model 1*		Model 2†	
			HR (95% CI)	*P* value	HR (95% CI)	*P* value
**CMD status**						
No CMDs	46 368	896	1 (ref)		1 (ref)	
1 CMD	100 640	2375	1.08 (1.00–1.16)	0.063	1.07 (0.99–1.15)	0.099
Diabetes	1360	44	1.50 (1.11–2.04)	0.008	1.48 (1.09–2.00)	0.012
CHD	1996	77	1.52 (1.20–1.92)	< 0.001	1.50 (1.19–1.90)	< 0.001
Stroke	547	25	2.05 (1.37–3.04)	< 0.001	2.05 (1.38–3.05)	< 0.001
Hypertension	96 737	2229	1.05 (0.97–1.14)	0.19	1.05 (0.97–1.13)	0.26
2 CMDs	20 918	935	1.78 (1.62–1.96)	< 0.001	1.74 (1.58–1.91)	< 0.001
Diabetes and CHD	246	11	1.79 (0.99–3.25)	0.055	1.72 (0.95–3.12)	0.075
Diabetes and stroke	40	0				
Diabetes and hypertension	8638	384	1.94 (1.72–2.20)	< 0.001	1.88 (1.66–2.12)	< 0.001
CHD and stroke	90	12	4.40 (2.49–7.79)	< 0.001	4.14 (2.34–7.33)	< 0.001
CHD and hypertension	9590	404	1.57 (1.40–1.77)	< 0.001	1.54 (1.37–1.74)	< 0.001
Stroke and hypertension	2314	124	2.08 (1.72–2.51)	< 0.001	2.02 (1.68–2.45)	< 0.001
≥ 3 CMDs	3612	273	2.97 (2.58–3.41)	< 0.001	2.83 (2.46–3.25)	< 0.001
Diabetes, CHD, and stroke	15	1	4.44 (0.63–31.6)	0.14	4.27 (0.60–30.4)	0.15
Diabetes, CHD, and hypertension	2333	161	2.78 (2.35–3.30)	< 0.001	2.65 (2.23–3.14)	< 0.001
Diabetes, stroke, and hypertension	374	35	3.57 (2.54–5.02)	< 0.001	3.44 (2.45–4.83)	< 0.001
CHD, stroke, and hypertension	673	50	2.58 (1.94–3.44)	< 0.001	2.46 (1.85–3.28)	< 0.001
Diabetes, CHD, stroke, and hypertension	217	26	5.84 (3.95–8.63)	< 0.001	5.50 (3.72–8.14)	< 0.001
**Lifestyle category**						
Unfavourable	25 472	898	1 (ref)		1 (ref)	
Intermediate	90 102	2387	0.75 (0.70–0.81)	< 0.001	0.78 (0.72–0.84)	< 0.001
Favourable	55 964	1194	0.63 (0.57–0.68)	< 0.001	0.67 (0.61–0.73)	< 0.001

CHD: coronary heart disease; CMD, cardiometabolic disease; HR: hazard ratio

*HRs were estimated using Cox regression models with age as time scale and adjusted for sex, ethnicity, education, socioeconomic deprivation, depression, *APOE* ε4 carrier status, and cognitive performance at baseline (model 1)

†Additionally adjusted for lifestyle category or CMD status (model 2)

### Association of CMDs and lifestyle with dementia risk

**Fig. 1:**
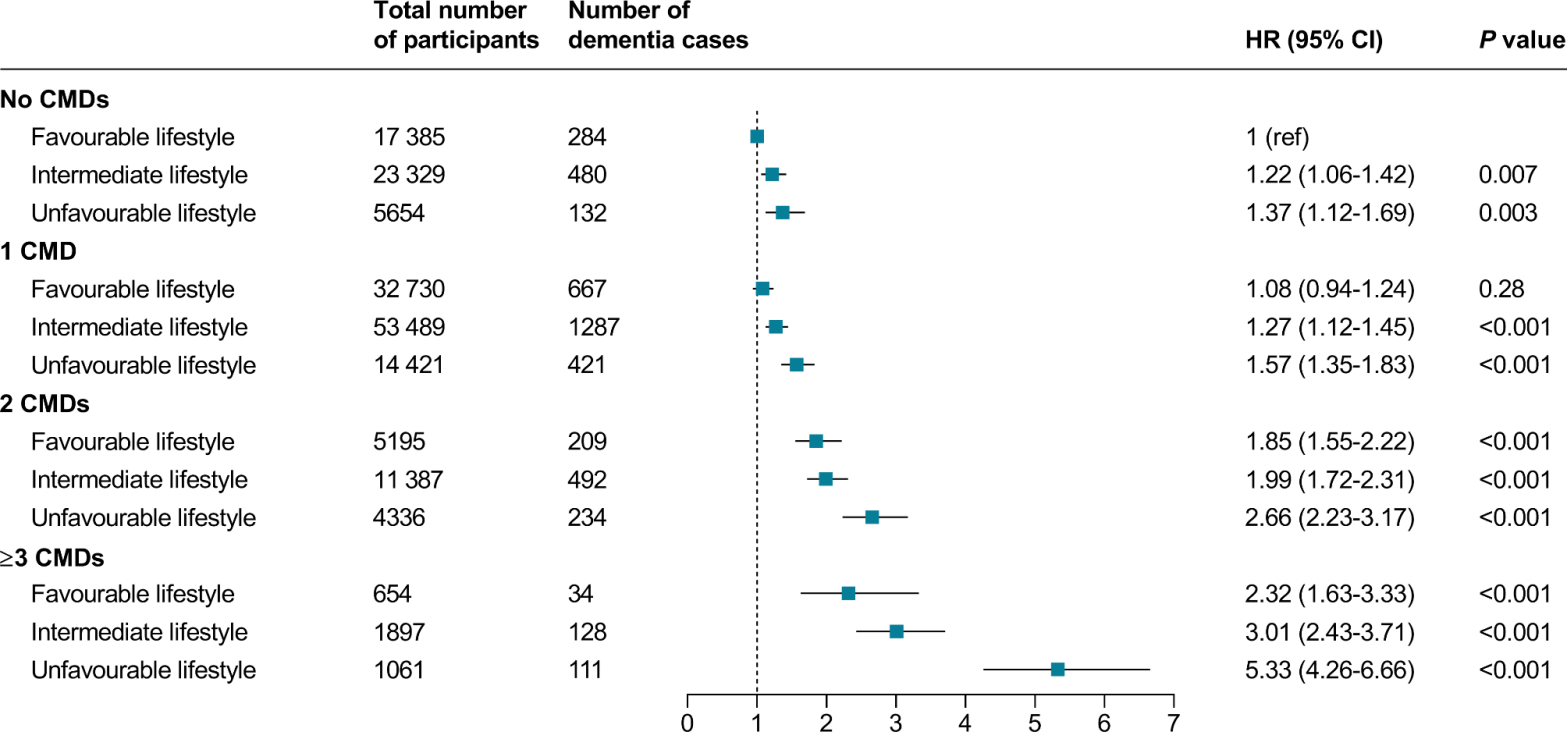
Risk of incident dementia according to cardiometabolic disease status and lifestyle category Cox regression models with age as time scale were adjusted for sex, ethnicity, education, socioeconomic deprivation, depression, *APOE* ε4 carrier status, and cognitive performance at baseline. The reference group was participants with no CMDs and a favourable lifestyle. Test for interaction between CMD status and lifestyle category was significant (*P*_interaction_=0.001). CMD: cardiometabolic disease; HR: hazard ratio

When CMD status and lifestyle profile were combined, the risk of incident dementia increased with an increasing number of CMDs and an increasingly unhealthy lifestyle (Fig. 1). Compared with participants with no CMDs and a favourable lifestyle, those with ≥ 3 CMDs and an unfavourable lifestyle had a 5-fold greater risk of dementia (HR 5.33, 95% CI 4.26–6.66). However, the risk was markedly reduced among participants with ≥ 3 CMDs and a favourable lifestyle (HR 2.32, 1.63–3.33). A significant interaction was observed between CMD status and lifestyle category on dementia (*P*_interaction_ = 0.001). Analyses of the dementia subtypes showed more pronounced results for vascular dementia compared to Alzheimer’s disease (Supplementary Table 5). The association of CMDs and lifestyle with dementia remained consistent in serial sensitivity analyses when using a weighted lifestyle score, redefining low-risk alcohol consumption as no heavy drinking, or excluding dementia cases during the first 3 or 5 years of follow-up (Supplementary Tables 6–8). Considering the competing risk of death and imputing missing data for exposure and covariates yielded similar results (Supplementary Tables 9–10). The HRs did not appreciably change after adjustment for upstream cardiometabolic risk markers and was slightly attenuated after adjustment for medications on metabolic disorders (Supplementary Table 11). Stratified analyses showed that the joint association of CMD status and lifestyle with dementia risk was stronger in *APOE* ε4 noncarriers than in *APOE* ε4 carriers (*P*_interaction_ <0.001) (Supplementary Table 12).

Within each CMD status, a favourable lifestyle was associated with a lower risk of developing dementia (Table 3). The HR for dementia comparing favourable lifestyle versus unfavourable lifestyle was 0.71 (95% CI 0.58–0.88) among participants with no CMDs and 0.42 (95% CI 0.28–0.63) among participants with ≥ 3 CMDs, with an absolute difference in incident dementia rates of − 0.65 (95% CI − 1.02 to − 0.27) and − 5.64 (95% CI − 8.11 to − 3.17) per 1000 person-years, respectively. A similar pattern was found for separate conventional and emerging lifestyle score although the HR estimates were less remarkable than the extended score (Supplementary Table 13).

**Table 3:** Incidence rates and risk of dementia according to lifestyle category within each cardiometabolic disease status

	Unfavourable lifestyle	Intermediate lifestyle	Favourable lifestyle
**No CMDs**			
Number of dementia cases/person-years	132/ 66 452	480/ 281 044	284/ 211 743
Incidence rate per 1000 person-years (95% CI)	1.99 (1.67–2.36)	1.71 (1.56–1.87)	1.34 (1.19–1.51)
Absolute rate difference per 1000 person-years (95% CI)	1 (ref)	−0.28 (− 0.65 to 0.09)	−0.65 (− 1.02 to − 0.27)
HR (95% CI)*	1 (ref)	0.87 (0.72–1.06)	0.71 (0.58–0.88)
*P* value		0.18	0.002
**1 CMD**			
Number of dementia cases/person-years	421/ 167 252	1287/ 636 725	667/ 392 382
Incidence rate per 1000 person-years (95% CI)	2.52 (2.29–2.77)	2.02 (1.91–2.14)	1.70 (1.58–1.83)
Absolute rate difference per 1000 person-years (95% CI)	1 (ref)	−0.50 (− 0.76 to − 0.23)	−0.82 (− 1.09 to − 0.54)
HR (95% CI)	1 (ref)	0.81 (0.73–0.91)	0.70 (0.61–0.79)
*P* value		< 0.001	< 0.001
**2 CMDs**			
Number of dementia cases/person-years	234/47 910	492/131 407	209/60 867
Incidence rate per 1000 person-years (95% CI)	4.88 (4.30–5.55)	3.74 (3.43–4.09)	3.43 (3.00–3.93)
Absolute rate difference per 1000 person-years (95% CI)	1 (ref)	−1.14 (− 1.85 to − 0.43)	−1.45 (− 2.23 to − 0.67)
HR (95% CI)	1 (ref)	0.76 (0.65–0.89)	0.71 (0.58–0.86)
*P* value		< 0.001	< 0.001
≥ 3 CMDs			
Number of dementia cases/person-years	111/10 787	128/20 684	34/7312
Incidence rate per 1000 person-years (95% CI)	10.3 (8.54–12.4)	6.19 (5.20–7.36)	4.65 (3.32–6.51)
Absolute rate difference per 1000 person-years (95% CI)	1 (ref)	−4.10 (− 6.30 to − 1.91)	−5.64 (− 8.11 to − 3.17)
HR (95% CI)	1 (ref)	0.57 (0.44–0.74)	0.42 (0.28–0.63)
*P* value		< 0.001	< 0.001

CMD: cardiometabolic disease; HR: hazard ratio

*Cox regression models with age as time scale were adjusted for sex, ethnicity, education, socioeconomic deprivation, depression, *APOE* ε4 carrier status, and cognitive performance at baseline. Participants in the unfavourable lifestyle category within each CMD status served as the reference group

### Association of CMDs and lifestyle with brain structural measures

**Fig. 2:**
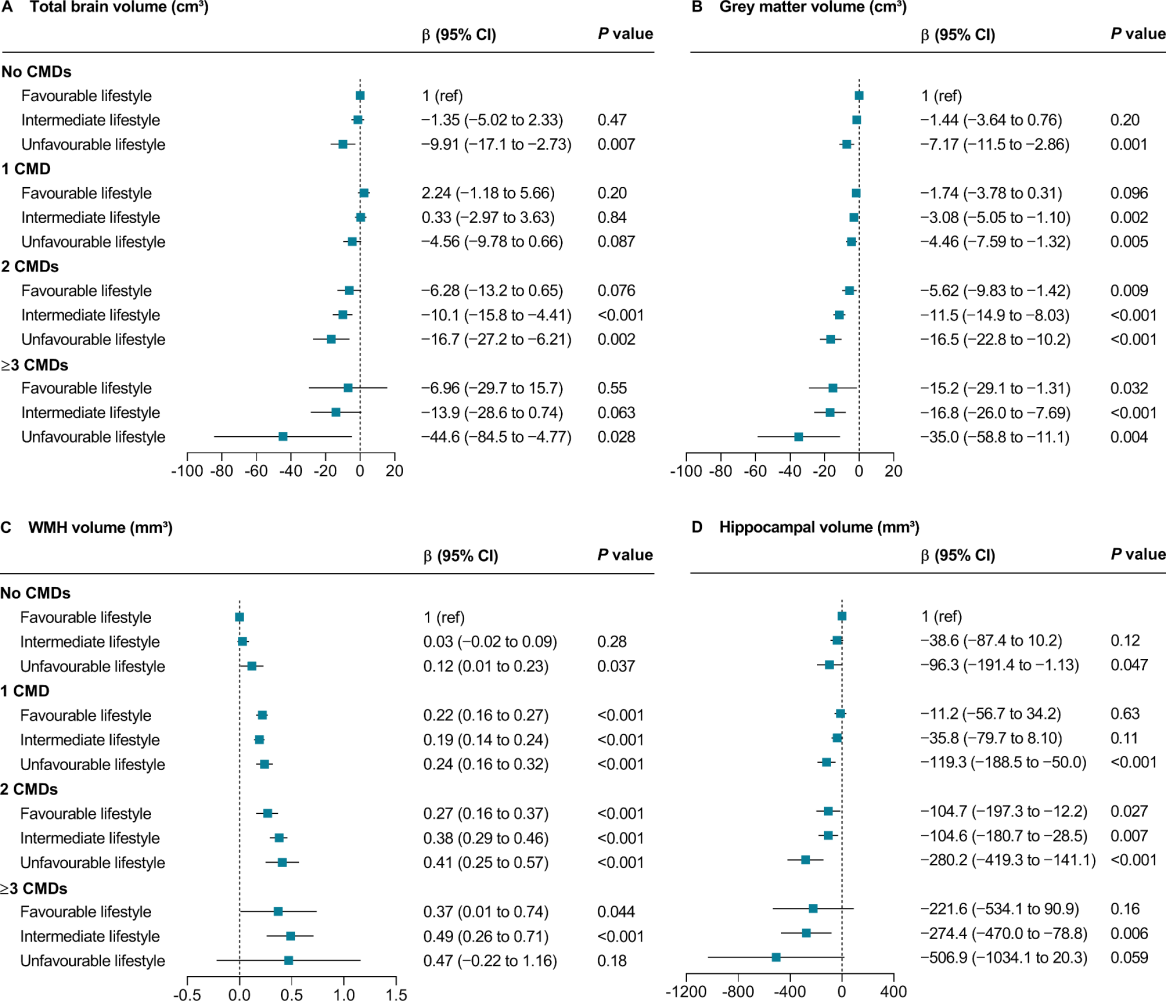
Associations of cardiometabolic disease status and lifestyle category with structural brain volumes Coefficients for differences in total brain volume (A), grey matter volume (B), WHM volume (C), and hippocampal volume (D) were estimated using linear regression models and adjusted for age, sex, ethnicity, education, socioeconomic deprivation, depression, *APOE* ε4 carrier status, and cognitive performance at baseline. The reference group was participants with no CMDs and a favourable lifestyle. WMH was log-transformed in the analysis due to its skewed distribution. Interaction between CMD status and lifestyle category: *P*_interaction_=0.26 for total brain volume, *P*_interaction_=0.23 for grey matter volume, *P*_interaction_=0.63 for WMH volume, *P*_interaction_=0.41 for hippocampal volume. CMD: cardiometabolic disease; WMH: white matter hyperintensity

In an imaging subsample of 11 978 participants, cardiometabolic multimorbidity and unfavourable lifestyle were each associated with smaller volumes of total brain, grey matter, and hippocampus and higher WMH load (Supplementary Table 14). When considered jointly, compared with participants who had no CMDs and a favourable lifestyle, participants with ≥ 3 CMDs and an unfavourable lifestyle had 44.6 cm^3^ lower total brain volume (*P* = 0.028), 35.0 cm^3^ lower grey matter volume (*P* = 0.004), and 506.9 mm^3^ lower hippocampal volume (*P* = 0.059); however, the volume reductions were largely mitigated in those with a favourable lifestyle (Fig. 2). We identified no significant interaction between CMD status and lifestyle category on brain structural measures. The results remained similar when the inverse probability weighting method was applied (Supplementary Table 15).

## Discussion

This prospective study provided quantitative data about the interplay between CMD status and lifestyle factors for dementia incidence. Cardiometabolic multimorbidity and overall lifestyle defined by seven lifestyle factors were independently associated with dementia risk. Within any CMD status, a favourable lifestyle was associated with a significantly lower risk of dementia. This association was stronger in participants with cardiometabolic multimorbidity than in those with no or single CMD. The results were corroborated in analyses using brain MRI data for a subcohort. A favourable lifestyle was associated with higher brain volumes, irrespective of CMD status.

Type 2 diabetes, CHD, and stroke are well-recognised risk factors for dementia, with each being associated with up to a two times increased risk of developing dementia [1–3]. Despite the fact that cardiometabolic multimorbidity is highly prevalent in people living with dementia [9], few studies have examined the association between multiple cardiometabolic conditions and dementia, and most were limited by small sample size. In the cohort of 2648 older adults from the Swedish National Study on Aging and Care-Kungsholmen (SNAC-K), type 2 diabetes, CHD, and stroke in combination was associated with a HR of 4.76 (95% CI 2.04–11.1) for dementia [15]. A subsequent analysis based on the same cohort reported that individuals with cardiometabolic multimorbidity had nearly twice the risk of cognitive impairment and its progression to dementia compared with those who were CMD-free [7]. Recently, a large study used data from 203 038 UK Biobank participants of European ancestry to investigate the association of stroke, myocardial infarction, and diabetes with dementia risk, and has shown an increase in HRs from 1.94 (95% CI 1.80–2.08) for one CMD to 5.55 (3.39–9.08) for three CMDs [8]. However, type 1 diabetes was not distinguished in this study and undiagnosed diabetes was not considered. In the current study that examined stroke, CHD, hypertension and type 2 diabetes that included both diagnosed and undiagnosed cases, participants with more CMDs had an increasingly higher risk of dementia after accounting for *APOE* ε4 allele and lifestyle factors; the highest HR (5.61) was observed for participants with four CMDs, suggesting a monotonic, additive relationship between increasing number of CMDs and dementia risk. This finding emphasises the importance of prevention of comorbid CMDs and aggressive intervention actions among patients who already have cardiometabolic multimorbidity to reduce the burden of dementia.

There is considerable evidence that healthy lifestyle factors such as no smoking, moderate alcohol consumption, regular physical exercise, and healthy diet could individually contribute to a lower risk of dementia [9]. Combination of lifestyle factors may interact synergistically and result in stronger associations with dementia risk [10, 11]. However, previous studies have mostly focused on the general population, little is known about the impact of a wide range of lifestyle factors on dementia incidence in people with CMDs. Results from the SNAC-K study showed a reduced risk of dementia associated with high levels of leisure activities and a rich social network in older adults with CMDs [15]. In the present study, we created a lifestyle score incorporating traditional (smoking, alcohol consumption, physical activity, and diet) and emerging lifestyle factors including sleep duration, sedentary behaviour, and social contact. Compared with participants with no CMDs and a favourable lifestyle, we found a five times increased risk of dementia in participants with ≥ 3 CMDs and an unfavourable lifestyle, but the risk was lowered by approximately 68% in those with a favourable lifestyle. Our study adds to the existing knowledge by demonstrating that the increased risk of dementia due to cardiometabolic multimorbidity might be offset by adherence to a healthy lifestyle.

Although favourable lifestyle was associated with a decreased risk of dementia within each CMD status, the risk reductions were more prominent in individuals with cardiometabolic multimorbidity than in those with no CMDs or only one CMD. The results imply that individuals with cardiometabolic multimorbidity might benefit more than others from adopting a healthy lifestyle. Several trials have also shown benefits of multidomain lifestyle interventions on improving cognitive function in older people with an elevated risk of dementia [31–33]. Therefore, from a public health perspective, lifestyle modification could be a feasible and effective prevention strategy that is likely to have a significant impact on dementia risk reduction in people with CMDs, particularly those with cardiometabolic multimorbidity.

Total and regional brain volumes are usually recognised as reliable predictors of dementia. For example, total hippocampal volume loss is associated with accelerated memory decline that predicts Alzheimer’s disease and grey matter measures could be used to track Alzheimer’s disease progression [34, 35], while higher WMH load is a major contributor to vascular cognitive impairment [36]. Consistent with previous studies reporting that CMDs were associated with smaller volumes of total brain, grey matter, and hippocampal and higher WMH load [16, 17], our findings showed graded associations between the number of CMDs and widespread negative brain changes. Compared with participants with no CMDs and a favourable lifestyle, the reductions in brain volumes were most significant among individuals with cardiometabolic multimorbidity and an unfavourable lifestyle but were largely attenuated among those who had a favourable lifestyle, suggesting that adherence to a healthy lifestyle might mitigate the deleterious impact of comorbid CMDs on structural brain health.

The mechanisms underpinning the putative protective effect of healthy lifestyle on dementia are not fully understood. Several hypotheses have been proposed involving inhibition of oxidative stress and inflammation, increase of cerebral blood flow, and reduction of amyloid aggregation and neuritic plaques [37], which highlight the role of healthy lifestyle in improving vascular and neurodegenerative pathologies, while cardiometabolic multimorbidity has been shown to cause global cerebrovascular and neurodegenerative processes that contribute to cognitive impairment and dementia [8, 38]. Furthermore, a healthy lifestyle may help patients with CMDs to better cope with medical resources, health behaviours (e.g. adhering to disease treatment, screening for complications), or psychosocial factors [37, 39]. All these aspects presumably favor brain integrity and function, thereby reducing dementia risk.

To our knowledge, this study is the first to examine the association of combined cardiometabolic multimorbidity and lifestyle with dementia incidence. Strengths of this study include use of a large, longitudinal cohort with a long follow up, utilization of comprehensive information to determine an extended lifestyle score combining conventional and emerging factors, and inclusion of data on brain imaging. Some limitations should be discussed. First, lifestyle behaviours were self-reported and misclassification of exposure was inevitable. However, such misclassification errors most likely lead to biased findings toward the null and underestimated the strength of the observed association between lifestyle and dementia risk. Besides, lifestyle metrics were derived from baseline data, and possible changes over time could not be accounted for. Second, UK Biobank participants are more likely to be from less deprived areas and have fewer health conditions such as cardiovascular disease than the general population [40]. Consequently, the effects of cardiometabolic risk and lifestyle could potentially be larger in more representative cohorts due to this selection bias. Third, although the large sample facilitates the investigation of CMD status and lifestyle in combination, certain study groups have low numbers, which might lead to a potential bias. Fourth, the subgroup of individuals with imaging data was generally a little younger, more educated, and less deprived. Therefore, the findings with brain volumes might not be generalised to the whole UK population. However, analyses after weighting by the inverse of probability of attending imaging test yielded similar results. Fifth, because of the observational nature of this study, reverse causality is still possible, even though the results were similar after excluding events occurring in the first 3 or 5 years of follow-up. Given the long preclinical phase before any evident symptoms of dementia, participants who developed dementia might be already in the preclinical phase of the disease or experience brain structural changes. Further prospective studies with a longer follow-up period are essential to validate our findings. Finally, although we adjusted for various potential confounders, residual confounding from unknown or unmeasured factors could not be ruled out.

## Conclusion

Our study indicated that cardiometabolic multimorbidity was associated with an increased risk of dementia and reduced brain volumes, while adherence to a healthy lifestyle might attenuate the dementia risk and adverse brain structural changes associated with cardiometabolic multimorbidity. These findings provide strong evidence supporting both clinical guidance and public health initiatives to promote an overall healthy lifestyle to prevent or delay the onset of dementia in people with cardiometabolic multimorbidity.

### Electronic supplementary material

Below is the link to the electronic supplementary material.

Supplementary Material 1

## Data Availability

The data used in this current study are available from the UK Biobank data resources. Permissions are required in order to gain access to the UK Biobank data resources, subject to successful registration and application process. Further information can be found on the UK Biobank website (https://www.ukbiobank.ac.uk/).

## Abbreviations

APOE: apolipoprotein E
BMI: body mass index
CHD: coronary heart disease
CI: confidence interval
CMD: cardiometabolic disease
ICD: International Classification of Diseases
HbA1c: glycated hemoglobin
HR: hazard ratio
WMH: white matter hyperintensity
