# Supplementary material for: Association of cardiometabolic multimorbidity and adherence to a healthy lifestyle with incident dementia: a large prospective cohort study
Source: Diabetol Metab Syndr. 2023 Oct 24;15:208. doi: 10.1186/s13098-023-01186-8 (PMC10594816; doi:10.1186/s13098-023-01186-8)
Supplement: Supplementary file 1 — Supplementary Material 1 [file 13098_2023_1186_MOESM1_ESM.docx]

**Supplementary Online Content**

**Association of cardiometabolic multimorbidity and adherence to a healthy lifestyle with incident dementia: a large prospective cohort study**

**Supplementary Table 1**. Codes used in the UK Biobank to identify dementia and cardiometabolic disease cases.

**Supplementary Table 2**. Behavioral lifestyle factor definitions in the UK Biobank study.

**Supplementary Table 3**. Associations between individual lifestyle factors and risk of incident dementia.

**Supplementary Table 4**. Association between the number of healthy lifestyle factors and incident dementia.

**Supplementary Table 5**. Risk of Alzheimer’s disease and vascular dementia according to cardiometabolic disease status and lifestyle category

**Supplementary Table 6**. Risk of incident dementia according to cardiometabolic disease status and lifestyle category based on weighted lifestyle score.

**Supplementary Table 7**. Risk of incident dementia according to cardiometabolic disease status and lifestyle category, redefining low-risk alcohol consumption as no heavy drinking.

**Supplementary Table 8**. Risk of incident dementia according to cardiometabolic disease status and lifestyle category after excluding incident cases occurring during the first 3 or 5 years of follow-up.

**Supplementary Table 9**. Risk of incident dementia according to cardiometabolic disease status and lifestyle category using competing risk regression analysis.

**Supplementary Table 10**. Risk of incident dementia according to cardiometabolic disease status and lifestyle category using multiple imputation to assign missing values of exposure and covariates.

**Supplementary Table 11**. Risk of incident dementia according to cardiometabolic disease status and lifestyle category after additionally controlling for upstream risk factors or medications.

**Supplementary Table 12**. Risk of incident dementia according to cardiometabolic disease status and lifestyle category, stratified by sex, education and *APOE* ε4 status.

**Supplementary Table 13**. Risk of incident dementia according to conventional and emerging lifestyle factors by cardiometabolic disease status.

**Supplementary Table 14**. Associations of cardiometabolic disease status and lifestyle with brain volumes.

**Supplementary Table 15**. Associations of cardiometabolic disease status and lifestyle with brain volumes using the inverse probability weighting method.

**Supplementary Figure 1**. Study flow diagram.

| **Supplementary Table 1**. Codes used in the UK Biobank to identify dementia and cardiometabolic disease cases. | | | |
| --- | --- | --- | --- |
|  | **Self-reported** | **ICD-9 codes** | **ICD-10 codes** |
| **Dementia** | 1263 | Alzheimer’s disease: 331.0  Vascular dementia: 290.4 Frontotemporal dementia: 331.1  Other codes for all-cause dementia: 290.2, 290.3, 291.2, 294.1, 331.2, 331.5 | Alzheimer’s disease: F00, F00.0, F00.1, F00.2, F00.9, G30, G30.0, G30.1, G30.8, G30.9  Vascular dementia: F01, F01.0, F01.1, F01.2, F01.3, F01.8, F01.9, I67.3  Frontotemporal dementia: F02.0, G31.0  Other codes for all-cause dementia: A81.0, F02, F02.1, F02.2, F02.3, F02.4, F02.8, F03, F05.1, F10.6, G31.1, G31.8 |
|  |  |  |  |
|  |  |  |  |
|  |  |  |  |
|  |  |  |  |
| **Diabetes** | 2443, 20002 (1220, 1223), 20003, 6177 (3), 6153 (3) | 250 | E11 |
| **Coronary heart disease** | 6150 (1, 2), 20002 (1074, 1075) | 410-414 | I20-I25 |
| **Stroke** | 6150 (3), 20002 (1081, 1086, 1491, 1583) | 430-434, 436 | I60-I64 |
| **Hypertension** | 6150 (4), 20002 (1065, 1072), 6153 (2), 6177 (2) | 401-405 | I10-I13, I15, O10 |

| **Supplementary Table 2**. Behavioral lifestyle factor definitions in the UK Biobank study. | | | |
| --- | --- | --- | --- |
| **Lifestyle factor** | **Questionnaire** | **“Healthy” category** | **“Unhealthy” category** |
| **Smoking** | “Do you smoke tobacco now?” and “In the past, how often have you smoked tobacco?” | Past or never smoker | Current smoker |
| **Alcohol intake** | “About how often do you drink alcohol?” Depending on the reported frequency, participants were further asked how much specified alcohol they drank on average in a week or month^a^ | Up to 1 drink (14 g)/day for women and up to 2 drinks (28 g)/day for men | Nonregular (no drinking or drinking on special occasions) or heavy drinkers |
| **Physical activity** | “In a typical week, how many days did you do 10 minutes or more of moderate (or vigorous) physical activity?” and “How many minutes did you usually spend doing moderate (or vigorous) activities on a typical day?” | ≥150 min/week moderate or ≥75 min/week vigorous activity (or an equivalent combination) | <150 min/week moderate and <75 min/week vigorous activity |
| **Diet** | Food frequency questionnaire on 7 groups including fruits (fresh and dried), vegetables (cooked and salad/raw), fish (oily and non-oily), processed meats (e.g. bacon, ham, sausages, meat pies, kebabs, burgers, chicken nuggets), unprocessed meats (beef, lamb/mutton, and pork), whole grains (bread [wholemeal, wholegrain], cereal [bran, oat, muesli cereal]), refined grains (bread [white , brown, other], cereal [biscuit or other]) | At least 4 of the following 7 food groups^b^:  1. Fruits: ≥ 3 servings/day  2. Vegetables: ≥ 3 servings/day  3. (Shell)fish: ≥ 2 servings/week  4. Processed meats: ≤1 servings/week  5. Unprocessed meats: ≤2 serving/week  6. Whole grains: ≥3 servings/day  7. Refined grains: ≤2 servings/day | <4 of the 7 recommended food groups |
| **Sleep duration** | “About how many hours sleep do you get in every 24 hours? (Please include naps)” | 7–9 h/day | <7 or >9 h/day |
| **Television viewing time** | “In a typical day, how many hours do you spend watching TV?” | <4 h/day | ≥4 h/day |
| **Social contact** | Question 1: “how many people are living together in your household?” We assigned 1 point for reporting living alone.  Question 2: “How often do you visit friends or family or have them visit you?” We assigned 1 point for answering less than once a month.  Question 3: “Which of the following (sports club or gym, pub or social club, religious group, adult education class, other group activity) do you attend once a week or more often?” We assigned 1 point for answering none of the above. | Score ≤1 when individual scores were summed up to calculate an overall social score ranging from 0-3^c^ | Score ≥2 |

^a^Participants were asked the amount of red wine (glasses), champagne plus white wine (glasses), beer plus cider (pints), spirits (measures), fortified wine (glasses), and other alcoholic drinks (glasses) they consumed. Alcohol intake in units per day was calculated by summing the average individual drinks per day (1). The units were converted to grams assuming that one unit equals 8 g. Moderate alcohol consumption was defined with the maximum limit reflecting US dietary guidelines (2).

^b^We defined healthy diet as an adequate intake of at least 4 of the 7 dietary components including increased consumption of fruits, vegetables, whole grains, (shell)fish, and reduced or no consumption of refined grains, processed and unprocessed meats following recommendations on dietary priorities for cardiometabolic health (3,4).

^c^According to a previous UK Biobank study (5), the social score ranging from 0 to 3 based on three questions was calculated and categorised as active (score=0), moderately active (score=1), and isolated (scores≥2).

**References**

1. Alcohol units. Available from: https://www.nhs.uk/live-well/alcohol-support/calculating-alcohol-units/.

2. US Department of Health and Human Services. 2015-2020 Dietary guidelines for Americans. 8th edition. <https://health.gov/dietaryguidelines/2015/> resources/2015-2020_Dietary_Guidelines.pdf.

3. Lourida I, Hannon E, Littlejohns TJ, Langa KM, Hyppönen E, Kuzma E, et al. Association of lifestyle and genetic risk with incidence of dementia. JAMA. 2019;322:430-437.

4. Mozaffarian D. Dietary and policy priorities for cardiovascular disease, diabetes, and obesity: a comprehensive review. Circulation. 2016;133:187-225.

5. Smith RW, Barnes I, Green J, Reeves GK, Beral V, Floud S. Social isolation and risk of heart disease and stroke: analysis of two large UK prospective studies. Lancet Public Health. 2021:6:e232-e239

| **Supplementary Table 3**. Associations between individual lifestyle factors and risk of incident dementia. | | | | | | |
| --- | --- | --- | --- | --- | --- | --- |
|  | **Model 1^a^** | | **Model 2^b^** | | **Model 3^c^** | |
|  | **HR (95% CI)** | ***P* value** | **HR (95% CI)** | ***P* value** | **HR (95% CI)** | ***P* value** |
| **No current smoking** | 0.70 (0.63-0.77) | <0.001 | 0.82 (0.74-0.91) | <0.001 | 0.81 (0.73-0.90) | <0.001 |
| **Moderate alcohol consumption** | 0.83 (0.78-0.88) | <0.001 | 0.86 (0.81-0.91) | <0.001 | 0.87 (0.82-0.93) | <0.001 |
| **Regular physical activity** | 0.86 (0.81-0.91) | <0.001 | 0.87 (0.82-0.92) | <0.001 | 0.90 (0.85-0.95) | <0.001 |
| **Healthy diet** | 0.94 (0.88-0.99) | 0.033 | 1.04 (0.98-1.11) | 0.20 | 1.04 (0.98-1.10) | 0.23 |
| **Adequate sleep duration** | 0.82 (0.77-0.87) | <0.001 | 0.86 (0.81-0.92) | <0.001 | 0.88 (0.82-0.94) | <0.001 |
| **Less sedentary behavior** | 0.77 (0.73-0.82) | <0.001 | 0.83 (0.78-0.88) | <0.001 | 0.87 (0.81-0.92) | <0.001 |
| **Frequent social contact** | 0.63 (0.58-0.69) | <0.001 | 0.74 (0.67-0.81) | <0.001 | 0.75 (0.68-0.82) | <0.001 |

HR, hazard ratio.

^a^Model 1: unadjusted model.

^b^Model 2: Cox regression models with age as time scale were adjusted for sex, ethnicity, education, socioeconomic deprivation, depression, *APOE* ε4 carrier status, and cognitive performance at baseline. Individual lifestyle factors were mutually adjusted.

^c^Additionally adjusted for cardiometabolic disease status.

| **Supplementary Table 4**. Association between the number of healthy lifestyle factors and risk of incident dementia. | | | | | | |
| --- | --- | --- | --- | --- | --- | --- |
|  | **Total No. of**  **participants** | **No. of dementia**  **cases** | **Model 1^a^** | | **Model 2^b^** | |
|  |  |  | **HR (95% CI)** | ***P* value** | **HR (95% CI)** | ***P* value** |
| **No. of healthy lifestyle factors** |  |  |  |  |  |  |
| 0-1 | 1269 | 57 | 1 (ref) |  | 1 (ref) |  |
| 2 | 5625 | 236 | 0.83 (0.62-1.11) | 0.21 | 0.84 (0.63-1.13) | 0.25 |
| 3 | 18 578 | 605 | 0.64 (0.49-0.84) | 0.001 | 0.67 (0.51-0.88) | 0.004 |
| 4 | 38 519 | 1092 | 0.56 (0.43-0.73) | <0.001 | 0.59 (0.45-0.77) | <0.001 |
| 5 | 51 583 | 1295 | 0.50 (0.38-0.65) | <0.001 | 0.54 (0.41-0.71) | <0.001 |
| 6 | 40 706 | 914 | 0.46 (0.35-0.60) | <0.001 | 0.50 (0.38-0.66) | <0.001 |
| 7 | 15 258 | 280 | 0.38 (0.28-0.50) | <0.001 | 0.42 (0.31-0.56) | <0.001 |
| *P* for trend |  |  | <0.001 | | <0.001 | |
| Per 1-number increase |  |  | 0.87 (0.85-0.89) | <0.001 | 0.89 (0.87-0.91) | <0.001 |

HR, hazard ratio.

^a^Cox regression models with age as time scale were adjusted for sex, ethnicity, education, socioeconomic deprivation, depression, *APOE* ε4 carrier status, and cognitive performance at baseline (model 1).

^b^Additionally adjusted for cardiometabolic disease status.

| **Supplementary Table 5**. Risk of Alzheimer’s disease and vascular dementia according to cardiometabolic disease status and lifestyle category. | | | | | | | |
| --- | --- | --- | --- | --- | --- | --- | --- |
|  |  | **Vascular dementia** | | | **Alzheimer’s disease** | | |
|  | **Total No. of**  **participants** | **No. of**  **cases** | **HR (95% CI)^a^** | ***P* value** | **No. of**  **cases** | **HR (95% CI)^a^** | ***P* value** |
| **No CMDs** |  |  |  |  |  |  |  |
| Favourable lifestyle | 17 385 | 47 | 1 (ref) |  | 121 | 1 (ref) |  |
| Intermediate lifestyle | 23 329 | 71 | 1.05 (0.73-1.52) | 0.79 | 222 | 1.32 (1.06-1.65) | 0.013 |
| Unfavourable lifestyle | 5654 | 22 | 1.29 (0.77-2.14) | 0.33 | 48 | 1.18 (0.84-1.65) | 0.34 |
| **1 CMD** |  |  |  |  |  |  |  |
| Favourable lifestyle | 32 730 | 120 | 1.13 (0.81-1.59) | 0.48 | 294 | 1.11 (0.90-1.37) | 0.33 |
| Intermediate lifestyle | 53 489 | 251 | 1.40 (1.02-1.91) | 0.036 | 544 | 1.26 (1.04-1.54) | 0.021 |
| Unfavourable lifestyle | 14 421 | 72 | 1.46 (1.01-2.12) | 0.044 | 167 | 1.49 (1.17-1.88) | 0.001 |
| **2 CMDs** |  |  |  |  |  |  |  |
| Favourable lifestyle | 5195 | 64 | 3.14 (2.15-4.59) | <0.001 | 83 | 1.74 (1.31-2.31) | <0.001 |
| Intermediate lifestyle | 11 387 | 133 | 2.91 (2.07-4.08) | <0.001 | 170 | 1.62 (1.28-2.06) | <0.001 |
| Unfavourable lifestyle | 4336 | 75 | 4.50 (3.10-6.52) | <0.001 | 70 | 1.89 (1.40-2.55) | <0.001 |
| **≥3 CMDs** |  |  |  |  |  |  |  |
| Favourable lifestyle | 654 | 14 | 5.15 (2.82-9.40) | <0.001 | 11 | 1.78 (0.96-3.31) | 0.068 |
| Intermediate lifestyle | 1897 | 49 | 6.04 (4.02-9.07) | <0.001 | 45 | 2.53 (1.79-3.58) | <0.001 |
| Unfavourable lifestyle | 1061 | 41 | 9.84 (6.41-15.1) | <0.001 | 31 | 3.54 (2.37-5.28) | <0.001 |

CMD, cardiometabolic disease; HR, hazard ratio.

^a^Cox regression models with age as time scale were adjusted for sex, ethnicity, education, socioeconomic deprivation, depression, *APOE* ε4 carrier status, and cognitive performance at baseline. The reference group was participants with no CMDs and a favourable lifestyle.

*P* value for interaction between CMD status and lifestyle category was 0.049 on vascular dementia and 0.47 on Alzheimer’s disease.

| **Supplementary Table 6**. Risk of incident dementia according to cardiometabolic disease status and lifestyle category based on weighted lifestyle score^a^. | | | | |
| --- | --- | --- | --- | --- |
|  |  |  |  |  |
|  | **Total No. of**  **participants** | **No. of dementia**  **cases** | **HR (95% CI)^b^** | ***P* value** |
| **No CMDs** |  |  |  |  |
| Favourable lifestyle | 15 440 | 245 | 1 (ref) |  |
| Intermediate lifestyle | 24 790 | 498 | 1.22 (1.05-1.43) | 0.010 |
| Unfavourable lifestyle | 6138 | 153 | 1.49 (1.22-1.83) | <0.001 |
| **1 CMD** |  |  |  |  |
| Favourable lifestyle | 28 383 | 550 | 1.07 (0.92-1.24) | 0.41 |
| Intermediate lifestyle | 57 157 | 1389 | 1.31 (1.14-1.50) | <0.001 |
| Unfavourable lifestyle | 15 100 | 436 | 1.57 (1.34-1.84) | <0.001 |
| **2 CMDs** |  |  |  |  |
| Favourable lifestyle | 4388 | 172 | 1.86 (1.53-2.27) | <0.001 |
| Intermediate lifestyle | 12 070 | 525 | 2.05 (1.76-2.39) | <0.001 |
| Unfavourable lifestyle | 4460 | 238 | 2.69 (2.24-3.23) | <0.001 |
| **≥3 CMDs** |  |  |  |  |
| Favourable lifestyle | 533 | 28 | 2.47 (1.67-3.66) | <0.001 |
| Intermediate lifestyle | 1974 | 134 | 3.08 (2.49-3.82) | <0.001 |
| Unfavourable lifestyle | 1105 | 111 | 5.21 (4.15-6.55) | <0.001 |

CMD, cardiometabolic disease; HR, hazard ratio.

^a^The weighted lifestyle score was categorised into favourable, intermediate, and unfavourable lifestyle based on the distribution of the unweighted lifestyle score.

^b^Cox regression models with age as time scale were adjusted for sex, ethnicity, education, socioeconomic deprivation, depression, *APOE* ε4 carrier status, and cognitive performance at baseline. The reference group was participants with no CMDs and a favourable lifestyle.

| **Supplementary Table 7**. Risk of incident dementia according to cardiometabolic disease status and lifestyle category, redefining low-risk alcohol consumption as no heavy drinking. | | | | |
| --- | --- | --- | --- | --- |
|  |  |  |  |  |
|  | **Total No. of**  **participants** | **No. of dementia**  **cases** | **HR (95% CI)^a^** | ***P* value** |
| **No CMDs** |  |  |  |  |
| Favourable lifestyle | 19 951 | 346 | 1 (ref) |  |
| Intermediate lifestyle | 22 232 | 460 | 1.16 (1.01-1.34) | 0.033 |
| Unfavourable lifestyle | 4185 | 90 | 1.21 (0.96-1.53) | 0.11 |
| **1 CMD** |  |  |  |  |
| Favourable lifestyle | 37 937 | 801 | 1.05 (0.93-1.19) | 0.44 |
| Intermediate lifestyle | 51 696 | 1271 | 1.24 (1.10-1.39) | <0.001 |
| Unfavourable lifestyle | 11 007 | 303 | 1.42 (1.21-1.65) | <0.001 |
| **2 CMDs** |  |  |  |  |
| Favourable lifestyle | 6477 | 276 | 1.86 (1.59-2.19) | <0.001 |
| Intermediate lifestyle | 11 352 | 501 | 1.92 (1.67-2.21) | <0.001 |
| Unfavourable lifestyle | 3089 | 158 | 2.43 (2.01-2.95) | <0.001 |
| **≥3 CMDs** |  |  |  |  |
| Favourable lifestyle | 833 | 45 | 2.32 (1.70-3.18) | <0.001 |
| Intermediate lifestyle | 2014 | 154 | 3.22 (2.65-3.90) | <0.001 |
| Unfavourable lifestyle | 765 | 74 | 4.80 (3.72-6.19) | <0.001 |

CMD, cardiometabolic disease; HR, hazard ratio.

^a^Cox regression models with age as time scale were adjusted for sex, ethnicity, education, socioeconomic deprivation, depression, *APOE* ε4 carrier status, and cognitive performance at baseline. The reference group was participants with no CMDs and a favourable lifestyle.

| **Supplementary Table 8**. Risk of incident dementia according to cardiometabolic disease status and lifestyle category after excluding incident cases occurring during the first 3 or 5 years of follow-up. | | | | | | |
| --- | --- | --- | --- | --- | --- | --- |
|  |  |  |  |  |  |  |
|  | **Excluding dementia cases within 3**  **Years of follow-up** | | | **Excluding dementia cases within 5**  **Years of follow-up** | | |
|  | **No. of**  **cases** | **HR (95% CI)^a^** | ***P* value** | **No. of**  **cases** | **HR (95% CI)** | ***P* value** |
| **No CMDs** |  |  |  |  |  |  |
| Favourable lifestyle | 271 | 1 (ref) |  | 254 | 1 (ref) |  |
| Intermediate lifestyle | 456 | 1.22 (1.05-1.42) | 0.011 | 425 | 1.21 (1.04-1.42) | 0.015 |
| Unfavourable lifestyle | 118 | 1.29 (1.04-1.60) | 0.022 | 113 | 1.33 (1.06-1.66) | 0.013 |
| **1 CMD** |  |  |  |  |  |  |
| Favourable lifestyle | 643 | 1.09 (0.94-1.26) | 0.24 | 589 | 1.07 (0.92-1.24) | 0.39 |
| Intermediate lifestyle | 1227 | 1.27 (1.11-1.45) | <0.001 | 1108 | 1.23 (1.07-1.41) | 0.003 |
| Unfavourable lifestyle | 406 | 1.59 (1.36-1.85) | <0.001 | 377 | 1.59 (1.35-1.86) | <0.001 |
| **2 CMDs** |  |  |  |  |  |  |
| Favourable lifestyle | 197 | 1.83 (1.52-2.20) | <0.001 | 178 | 1.78 (1.47-2.16) | <0.001 |
| Intermediate lifestyle | 470 | 1.99 (1.71-2.32) | <0.001 | 438 | 2.01 (1.72-2.35) | <0.001 |
| Unfavourable lifestyle | 209 | 2.51 (2.09-3.01) | <0.001 | 191 | 2.49 (2.05-3.01) | <0.001 |
| **≥3 CMDs** |  |  |  |  |  |  |
| Favourable lifestyle | 30 | 2.17 (1.48-3.16) | <0.001 | 29 | 2.27 (1.54-3.34) | <0.001 |
| Intermediate lifestyle | 121 | 3.00 (2.42-3.73) | <0.001 | 111 | 3.00 (2.39-3.76) | <0.001 |
| Unfavourable lifestyle | 108 | 5.52 (4.40-6.92) | <0.001 | 95 | 5.33 (4.19-6.78) | <0.001 |

CMD, cardiometabolic disease; HR, hazard ratio.

^a^Cox regression models with age as time scale were adjusted for sex, ethnicity, education, socioeconomic deprivation, depression, *APOE* ε4 carrier status, and cognitive performance at baseline. The reference group was participants with no CMDs and a favourable lifestyle.

| **Supplementary Table 9**. Risk of incident dementia according to cardiometabolic disease status and lifestyle category using competing risk regression analysis. | | | | |
| --- | --- | --- | --- | --- |
|  |  |  |  |  |
|  | **Total No. of**  **participants** | **No. of dementia**  **cases** | **HR (95% CI)^a^** | ***P* value** |
| **No CMDs** |  |  |  |  |
| Favourable lifestyle | 17 385 | 284 | 1 (ref) |  |
| Intermediate lifestyle | 23 329 | 480 | 1.20 (1.04-1.39) | 0.014 |
| Unfavourable lifestyle | 5654 | 132 | 1.29 (1.05-1.59) | 0.017 |
| **1 CMD** |  |  |  |  |
| Favourable lifestyle | 32 730 | 667 | 1.08 (0.94-1.24) | 0.30 |
| Intermediate lifestyle | 53 489 | 1287 | 1.25 (1.10-1.42) | <0.001 |
| Unfavourable lifestyle | 14 421 | 421 | 1.47 (1.26-1.71) | <0.001 |
| **2 CMDs** |  |  |  |  |
| Favourable lifestyle | 5195 | 209 | 1.81 (1.51-2.17) | <0.001 |
| Intermediate lifestyle | 11 387 | 492 | 1.90 (1.64-2.21) | <0.001 |
| Unfavourable lifestyle | 4336 | 234 | 2.36 (1.98-2.82) | <0.001 |
| **≥3 CMDs** |  |  |  |  |
| Favourable lifestyle | 654 | 34 | 2.13 (1.49-3.06) | <0.001 |
| Intermediate lifestyle | 1897 | 128 | 2.70 (2.17-3.35) | <0.001 |
| Unfavourable lifestyle | 1061 | 111 | 4.36 (3.47-5.47) | <0.001 |

CMD, cardiometabolic disease; HR, hazard ratio.

^a^HRs were estimated using the Fine and Gray subdistribution hazard regression models with age as time scale after adjusting for sex, ethnicity, education, socioeconomic deprivation, depression, *APOE* ε4 carrier status, and cognitive performance at baseline. The reference group was participants with no CMDs and a favourable lifestyle.

| **Supplementary Table 10**. Risk of incident dementia according to cardiometabolic disease status and lifestyle category using multiple imputation by chained equations to assign missing values of exposure and covariates^a^. | | | | |
| --- | --- | --- | --- | --- |
|  |  |  |  |  |
|  | **Total No. of**  **participants** | **No. of dementia**  **cases** | **HR (95% CI)^b^** | ***P* value** |
| **No CMDs** |  |  |  |  |
| Favourable lifestyle | 17 720 | 292 | 1 (ref) |  |
| Intermediate lifestyle | 24 984 | 532 | 1.24 (1.08-1.44) | 0.003 |
| Unfavourable lifestyle | 6562 | 173 | 1.51 (1.24-1.84) | <0.001 |
| **1 CMD** |  |  |  |  |
| Favourable lifestyle | 37 185 | 808 | 1.12 (0.97-1.30) | 0.12 |
| Intermediate lifestyle | 64 128 | 1645 | 1.30 (1.15-1.49) | <0.001 |
| Unfavourable lifestyle | 19 051 | 635 | 1.68 (1.45-1.94) | <0.001 |
| **2 CMDs** |  |  |  |  |
| Favourable lifestyle | 5885 | 246 | 1.89 (1.58-2.26) | <0.001 |
| Intermediate lifestyle | 13 825 | 655 | 2.10 (1.82-2.43) | <0.001 |
| Unfavourable lifestyle | 5897 | 359 | 2.84 (2.41-3.34) | <0.001 |
| **≥3 CMDs** |  |  |  |  |
| Favourable lifestyle | 733 | 44 | 2.62 (1.87-3.66) | <0.001 |
| Intermediate lifestyle | 2358 | 166 | 3.10 (2.53-3.80) | <0.001 |
| Unfavourable lifestyle | 1472 | 155 | 5.17 (4.20-6.36) | <0.001 |

CMD, cardiometabolic disease; HR, hazard ratio.

^a^A multiple imputation approach by chained equations with 5 replicates was used to assign missing values for lifestyle factors and covariates. The estimates from 5 imputed data set were pooled with the use of Rubin’s rule. Predictors used in this imputation were all covariates, lifestyle factors, coronary heart disease, stroke, diabetes, hypertension, and body mass index.

^b^Cox regression models with age as time scale were adjusted for sex, ethnicity, education, socioeconomic deprivation, depression, *APOE* ε4 carrier status, and cognitive performance at baseline. The reference group was participants with no CMDs and a favourable lifestyle.

| **Supplementary Table 11**. Risk of incident dementia according to cardiometabolic disease status and lifestyle category after additionally controlling for upstream risk factors or medications. | | | | |
| --- | --- | --- | --- | --- |
|  |  |  |  |  |
|  | **Additionally adjusting for**  **cardiometabolic risk factors^a^** | | **Additionally adjusting for**  **medication use^b^** | |
|  | **HR (95% CI)** | ***P* value** | **HR (95% CI)** | ***P* value** |
| **No CMDs** |  |  |  |  |
| Favourable lifestyle | 1 (ref) |  | 1 (ref) |  |
| Intermediate lifestyle | 1.24 (1.07-1.43) | 0.005 | 1.22 (1.05-1.41) | 0.008 |
| Unfavourable lifestyle | 1.40 (1.13-1.72) | 0.002 | 1.37 (1.11-1.68) | 0.003 |
| **1 CMD** |  |  |  |  |
| Favourable lifestyle | 1.10 (0.95-1.26) | 0.20 | 1.03 (0.90-1.19) | 0.66 |
| Intermediate lifestyle | 1.31 (1.15-1.49) | <0.001 | 1.21 (1.06-1.38) | 0.005 |
| Unfavourable lifestyle | 1.63 (1.39-1.90) | <0.001 | 1.49 (1.27-1.74) | <0.001 |
| **2 CMDs** |  |  |  |  |
| Favourable lifestyle | 1.79 (1.49-2.16) | <0.001 | 1.67 (1.38-2.01) | <0.001 |
| Intermediate lifestyle | 1.96 (1.67-2.29) | <0.001 | 1.79 (1.53-2.10) | <0.001 |
| Unfavourable lifestyle | 2.65 (2.20-3.19) | <0.001 | 2.40 (1.99-2.89) | <0.001 |
| **≥3 CMDs** |  |  |  |  |
| Favourable lifestyle | 2.23 (1.55-3.20) | <0.001 | 2.08 (1.45-2.99) | <0.001 |
| Intermediate lifestyle | 2.93 (2.35-3.67) | <0.001 | 2.69 (2.16-3.35) | <0.001 |
| Unfavourable lifestyle | 5.27 (4.16-6.68) | <0.001 | 4.77 (3.78-6.01) | <0.001 |

CMD, cardiometabolic disease; HR, hazard ratio.

^a^Cox regression models with age as time scale were adjusted for sex, ethnicity, education, socioeconomic deprivation, depression, *APOE* ε4 carrier status, cognitive performance at baseline, total cholesterol, body mass index, and HbA_1c_ levels.

^b^Cox regression models with age as time scale were adjusted for sex, ethnicity, education, socioeconomic deprivation, depression, *APOE* ε4 carrier status, cognitive performance at baseline, and medications for high blood pressure, high cholesterol or diabetes.

| **Supplementary Table 12**. Risk of incident dementia according to cardiometabolic disease status and lifestyle category, stratified by sex, education and *APOE* ε4 status. | | | | | | | | | |
| --- | --- | --- | --- | --- | --- | --- | --- | --- | --- |
|  | **Women**  **(n =88 389)** | **Men**  **(n=83 149)** |  | **High education**^a^  **(n=47 323)** | **Low education**  **(n=122 805)** |  | **No *APOE* ε4**  **(n=122 616)** | **Any *APOE* ε4**  **(n=47 394)** |  |
|  | HR (95% CI)^b^ | HR (95% CI) | *P*_interaction_ | HR (95% CI) | HR (95% CI) | *P*_interaction_ | HR (95% CI) | HR (95% CI) | *P*_interaction_ |
| **No CMDs** |  |  |  |  |  |  |  |  |  |
| Favourable lifestyle | 1 (ref) | 1 (ref) | 0.87 | 1 (ref) | 1 (ref) | 0.80 | 1 (ref) | 1 (ref) | <0.001 |
| Intermediate lifestyle | 1.26 (1.03-1.54) | 1.17 (0.95-1.46) |  | 1.37 (1.05-1.78) | 1.14 (0.95-1.37) |  | 1.35 (1.08-1.69) | 1.14 (0.94-1.39) |  |
| Unfavourable lifestyle | 1.59 (1.21-2.10) | 1.13 (0.82-1.56) |  | 1.24 (0.74-2.07) | 1.36 (1.08-1.72) |  | 1.46 (1.07-2.00) | 1.34 (1.01-1.76) |  |
| **1 CMD** |  |  |  |  |  |  |  |  |  |
| Favourable lifestyle | 1.17 (0.96-1.41) | 0.99 (0.80-1.21) |  | 1.12 (0.88-1.43) | 1.04 (0.88-1.23) |  | 1.14 (0.92-1.42) | 1.03 (0.86-1.24) |  |
| Intermediate lifestyle | 1.31 (1.10-1.57) | 1.21 (1.01-1.46) |  | 1.34 (1.06-1.69) | 1.22 (1.04-1.42) |  | 1.37 (1.12-1.67) | 1.21 (1.02-1.43) |  |
| Unfavourable lifestyle | 1.56 (1.26-1.94) | 1.54 (1.24-1.91) |  | 1.51 (1.20-2.34) | 1.51 (1.26-1.81) |  | 1.90 (1.51-2.39) | 1.34 (1.09-1.65) |  |
| **2 CMDs** |  |  |  |  |  |  |  |  |  |
| Favourable lifestyle | 1.86 (1.37-2.51) | 1.77 (1.40-2.24) |  | 1.92 (1.37-2.69) | 1.79 (1.44-2.23) |  | 2.39 (1.84-3.11) | 1.50 (1.17-1.93) |  |
| Intermediate lifestyle | 2.20 (1.76-2.75) | 1.83 (1.49-2.24) |  | 1.97 (1.46-2.65) | 1.96 (1.64-2.34) |  | 2.64 (2.12-3.30) | 1.55 (1.26-1.90) |  |
| Unfavourable lifestyle | 2.79 (2.13-3.66) | 2.49 (1.96-3.16) |  | 2.83 (1.83-4.37) | 2.53 (2.07-3.10) |  | 3.70 (2.88-4.76) | 1.93 (1.49-2.49) |  |
| **≥3 CMDs** |  |  |  |  |  |  |  |  |  |
| Favourable lifestyle | 3.02 (1.54-5.93) | 2.03 (1.32-3.11) |  | 3.41 (1.77-6.56) | 1.92 (1.23-2.99) |  | 2.82 (1.67-4.77) | 2.04 (1.25-3.33) |  |
| Intermediate lifestyle | 4.59 (3.24-6.51) | 2.47 (1.88-3.24) |  | 4.15 (2.64-6.50) | 2.74 (2.14-3.50) |  | 4.12 (3.05-5.55) | 2.26 (1.67-3.07) |  |
| Unfavourable lifestyle | 5.74 (3.86-8.53) | 4.91 (3.71-6.51) |  | 8.88 (5.25-15.0) | 4.75 (3.69-6.12) |  | 6.64 (4.84-9.10) | 4.19 (3.02-5.81) |  |

CMD, cardiometabolic disease; HR, hazard ratio.

^a^High education indicates college or university degree.

^b^Cox regression models with age as time scale were adjusted for sex, ethnicity, education, socioeconomic deprivation, and depression, *APOE* ε4 carrier status, and cognitive performance at baseline. The strata variable was not included in the model when stratifying by itself. The reference group was participants with no CMDs and a favourable lifestyle.

| **Supplementary Table 13**. Risk of incident dementia according to conventional and emerging lifestyle factors by cardiometabolic disease status. | | | | | | | | |
| --- | --- | --- | --- | --- | --- | --- | --- | --- |
|  | **No CMDs** | | **1 CMD** | | **2 CMDs** | | **≥3 CMDs** | |
|  | **HR (95% CI)^a^** | ***P* value** | **HR (95% CI)** | ***P* value** | **HR (95% CI)** | ***P* value** | **HR (95% CI)** | ***P* value** |
| **Number of conventional**  **Healthy lifestyle factors^b^** |  |  |  |  |  |  |  |  |
| 0-1 | 1 (ref) |  | 1 (ref) |  | 1 (ref) |  | 1 (ref) |  |
| 2 | 0.96 (0.78-1.18) | 0.69 | 0.81 (0.72-0.92) | 0.001 | 0.97 (0.81-1.16) | 0.71 | 0.88 (0.65-1.19) | 0.40 |
| 3-4 | 0.80 (0.66-0.98) | 0.032 | 0.82 (0.73-0.92) | <0.001 | 0.84 (0.70-1.00) | 0.052 | 0.67 (0.48-0.92) | 0.012 |
| Per 1-number increment | 0.91 (0.85-0.98) | 0.011 | 0.93 (0.89-0.97) | <0.001 | 0.92 (0.86-0.99) | 0.020 | 0.86 (0.76-0.97) | 0.019 |
| **Number of emerging healthy**  **lifestyle factors^c^** |  |  |  |  |  |  |  |  |
| 0-1 | 1 (ref) |  | 1 (ref) |  | 1 (ref) |  | 1 (ref) |  |
| 2 | 0.84 (0.68-1.02) | 0.079 | 0.85 (0.76-0.95) | 0.005 | 0.86 (0.73-1.02) | 0.087 | 0.63 (0.48-0.83) | 0.001 |
| 3 | 0.74 (0.60-0.91) | 0.004 | 0.69 (0.61-0.78) | <0.001 | 0.79 (0.66-0.95) | 0.012 | 0.49 (0.34-0.68) | <0.001 |
| Per 1-number increment | 0.86 (0.79-0.94) | 0.001 | 0.84 (0.79-0.88) | <0.001 | 0.90 (0.82-0.98) | 0.014 | 0.72 (0.62-0.84) | <0.001 |

CMD, cardiometabolic disease; HR, hazard ratio.

^a^Cox regression models with age as time scale were adjusted for sex, ethnicity, education, socioeconomic deprivation, depression, *APOE* ε4 carrier status, and cognitive performance at baseline. Conventional and emerging lifestyle factors were mutually adjusted.

^b^Conventional healthy lifestyle factors include no current smoking, moderate alcohol consumption, regular physical activity, and healthy diet.

^c^Emerging healthy lifestyle factors include adequate sleep duration, less sedentary behavior, and frequent social contact.

|  | **Supplementary Table 14**. Associations of cardiometabolic disease status and lifestyle with brain volumes. | | | | | | | |
| --- | --- | --- | --- | --- | --- | --- | --- | --- |
|  | | **CMD status** | | | | **Lifestyle category** | | |
|  |  | **No CMDs** | **1 CMD** | **2 CMDs** | **≥3 CMDs** | **Unfavourable** | **Intermediate** | **Favourable** |
| **Total brain volume** | |  |  |  |  |  |  |  |
| No. of participants | | 4050 | 6757 | 943 | 94 | 1057 | 5903 | 4884 |
| Volume (cm^3^)^a^ | | 1446.0 (59.5) | 1445.7 (59.0) | 1431.3 (59.0) | 1427.5 (62.6) | 1440.5 (59.1) | 1444.4 (58.6) | 1445.4 (60.4) |
| β (95% CI)^b^ | | 0 (ref) | 2.22 (-0.04 to 4.48) | -7.76 (-11.9 to -3.62) | -12.9 (-24.7 to -1.14) | 0 (ref) | 6.32 (2.55 to 10.1) | 8.20 (4.33 to 12.1) |
| *P* value | |  | 0.054 | <0.001 | 0.032 |  | 0.001 | <0.001 |
| **Grey matter volume** | |  |  |  |  |  |  |  |
| No. of participants | | 4045 | 6720 | 921 | 88 | 1048 | 5864 | 4862 |
| Volume (cm^3^) | | 762.2 (36.5) | 757.4 (37.6) | 745.1 (37.4) | 737.2 (35.7) | 755.4 (37.6) | 757.3 (37.6) | 759.2 (37.3) |
| β (95% CI) | | 0 (ref) | -1.32 (-2.68 to 0.04) | -8.63 (-11.1 to -6.12) | -16.5 (-23.8 to -9.21) | 0 (ref) | 3.15 (0.88 to 5.42) | 4.84 (2.52 to 7.17) |
| *P* value | |  | 0.056 | <0.001 | <0.001 |  | 0.006 | <0.001 |
| **WMH volume^c^** | |  |  |  |  |  |  |  |
| No. of participants | | 3957 | 6529 | 900 | 85 | 1012 | 5720 | 4739 |
| Volume (mm^3^) | | 8.45 (0.85) | 8.65 (0.89) | 8.82 (0.90) | 8.90 (0.94) | 8.65 (0.91) | 8.60 (0.88) | 8.59 (0.88) |
| β (95% CI) | | 0 (ref) | 0.18 (0.14 to 0.21) | 0.32 (0.25 to 0.38) | 0.43 (0.25 to 0.62) | 0 (ref) | -0.06 (-0.12 to -0.003) | -0.06 (-0.12 to -0.004) |
| *P* value | |  | <0.001 | <0.001 | <0.001 |  | 0.038 | 0.037 |
| **Hippocampal volume** | |  |  |  |  |  |  |  |
| No. of participants | | 4016 | 6695 | 927 | 91 | 1052 | 5849 | 4828 |
| Volume (mm^3^) | | 7359.5 (790.3) | 7353.5 (789.1) | 7275.2 (815.2) | 7160.6 (840.7) | 7276.4 (777.8) | 7345.1 (786.7) | 7366.8 (801.5) |
| β (95% CI) | | 0 (ref) | -5.09 (-35.1 to 24.9) | -93.9 (-149.1 to -38.8) | -249.4 (-407.6 to -91.1) | 0 (ref) | 87.3 (37.3 to 137.3) | 114.9 (63.5 to 166.2) |
| *P* value | |  | 0.74 | <0.001 | 0.002 |  | <0.001 | <0.001 |

^a^Data are mean (SD). CMD, cardiometabolic disease; WMH, white matter hyperintensity.

^b^Linear regression models were adjusted for age, sex, ethnicity, education, socioeconomic deprivation, depression, *APOE* ε4 carrier status, and cognitive performance at baseline. CMD status and lifestyle category were mutually adjusted.

^c^WMH was log-transformed due to a skewed distribution.

| **Supplementary Table 15**. Associations of cardiometabolic disease status and lifestyle with brain volumes using the inverse probability weighting method^a^. | | | | | | | | |
| --- | --- | --- | --- | --- | --- | --- | --- | --- |
|  | **Total brain volume, cm^3^** | | **Grey matter volume, cm^3^** | | **WMH volume^b^, mm^3^** | | **Hippocampal volume, mm^3^** | |
|  | β (95% CI)^c^ | *P* value | β (95% CI) | *P* value | β (95% CI) | *P* value | β (95% CI) | *P* value |
| **No CMDs** |  |  |  |  |  |  |  |  |
| Favourable lifestyle | 0 (ref) |  | 0 (ref) |  | 0 (ref) |  | 0 (ref) |  |
| Intermediate lifestyle | 0.99 (-2.97 to 4.95) | 0.62 | -0.24 (-2.62 to 2.14) | 0.84 | 0.03 (-0.03 to 0.09) | 0.37 | -46.0 (-98.1 to 6.14) | 0.084 |
| Unfavourable lifestyle | -9.02 (-15.5 to -2.51) | 0.007 | -7.80 (-11.7 to -3.88) | <0.001 | 0.19 (0.09 to 0.29) | <0.001 | -157.3 (-243.2 to -71.4) | <0.001 |
| **1 CMD** |  |  |  |  |  |  |  |  |
| Favourable lifestyle | 2.46 (-1.39 to 6.30) | 0.21 | -1.17 (-3.48 to 1.14) | 0.32 | 0.21 (0.15 to 0.27) | <0.001 | -30.1 (-80.7 to 20.5) | 0.24 |
| Intermediate lifestyle | 1.88 (-1.69 to 5.45) | 0.30 | -2.17 (-4.32 to -0.02) | 0.048 | 0.18 (0.12 to 0.23) | <0.001 | -52.0 (-99.0 to -4.91) | 0.030 |
| Unfavourable lifestyle | -2.40 (-7.25 to 2.44) | 0.33 | -3.30 (-6.22 to -0.38) | 0.027 | 0.23 (0.16 to 0.30) | <0.001 | -139.7 (-203.3 to -76.1) | <0.001 |
| **2 CMDs** |  |  |  |  |  |  |  |  |
| Favourable lifestyle | -5.29 (-12.0 to 1.41) | 0.12 | -4.83 (-8.91 to -0.75) | 0.020 | 0.26 (0.15 to 0.36) | <0.001 | -133.6 (-222.1 to -45.2) | 0.003 |
| Intermediate lifestyle | -6.64 (-11.8 to -1.47) | 0.012 | -9.90 (-13.0 to -6.77) | <0.001 | 0.37 (0.29 to 0.45) | <0.001 | -112.8 (-181.1 to -44.5) | 0.001 |
| Unfavourable lifestyle | -18.4 (-26.4 to -10.4) | <0.001 | -17.8 (-22.7 to -12.9) | <0.001 | 0.51 (0.39 to 0.63) | <0.001 | -286.0 (-391.5 to -180.5) | <0.001 |
| **≥3 CMDs** |  |  |  |  |  |  |  |  |
| Favourable lifestyle | -13.8 (-32.1 to 4.46) | 0.14 | -17.8 (-29.2 to -6.52) | 0.002 | 0.43 (0.14 to 0.72) | 0.003 | -221.2 (-468.6 to 26.2) | 0.080 |
| Intermediate lifestyle | -10.4 (-21.2 to 0.24) | 0.055 | -16.0 (-22.7 to -9.39) | <0.001 | 0.53 (0.37 to 0.70) | <0.001 | -315.9 (-457.1 to -174.8) | <0.001 |
| Unfavourable lifestyle | -33.1 (-54.5 to -11.7) | 0.002 | -32.0 (-44.9 to -19.2) | <0.001 | 0.34 (-0.04 to 0.72) | 0.081 | -455.4 (-735.8 to -174.9) | 0.001 |

CMD, cardiometabolic disease; WMH, white matter hyperintensity.

^a^To account for the difference between participants with and without MRI data, we conducted sensitivity analysis using inverse probability weights for being included in brain MRI analysis. The probability of attending the brain MRI tests in the UK Biobank for each participant was calculated in logistic models that included terms for the covariates (see below) plus all 7 lifestyle factors, coronary heart disease, stroke, diabetes, and hypertension. In the linear regression models that tested the differences in brain volumes, individuals were weighted by the inverse of the probability of attending the brain MRI tests.

^b^WMH was log-transformed in the analysis due to its skewed distribution.

^c^Coefficients were estimated using linear regression models after adjusting for age, sex, ethnicity, education, socioeconomic deprivation, depression, *APOE* ε4 carrier status, and cognitive performance at baseline. The reference group was participants with no CMDs and a favourable lifestyle.

**Supplementary Figure 1**. Study flow diagram.

**
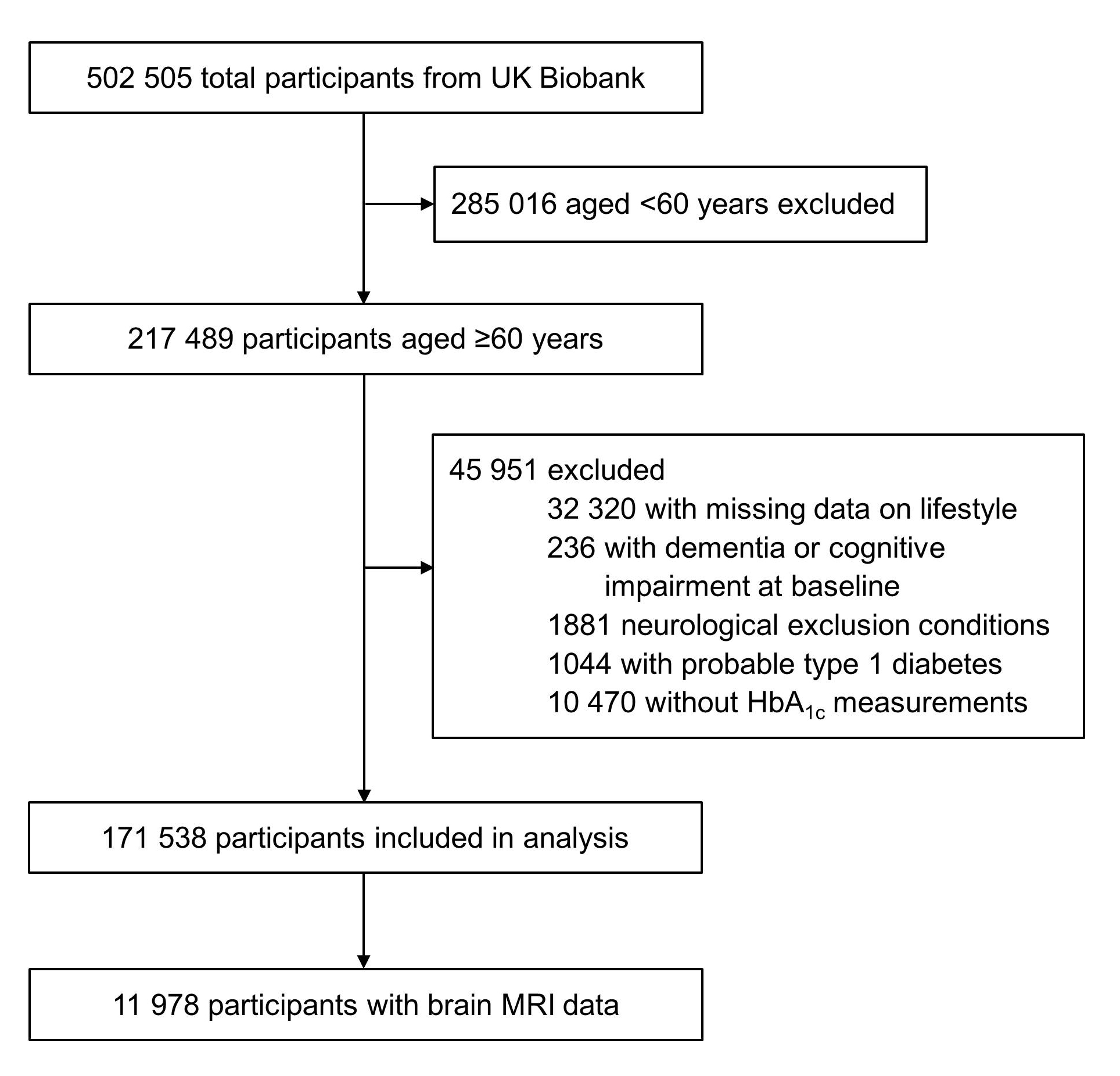
**
